# Prevalence and Characteristics of Delusional Infestation in Individuals With Opiate Use Disorders Within One Health System: A Retrospective Cross-Sectional Study

**DOI:** 10.7759/cureus.76098

**Published:** 2024-12-20

**Authors:** Manish Shrestha, Becky Cox-Davenport, Debra Powell, Anthony Donato

**Affiliations:** 1 Department of Medicine, Tower Health Medical Group, Reading, USA

**Keywords:** delusional parasitosis, fentanyl, illicit drugs, xylazine, substance abuse

## Abstract

Objective: The recent development of xylazine adulteration of the illicit opiate supply has been associated with necrotic extremity wounds of unclear pathogenesis. This study sought to understand the prevalence and characteristics of delusional infestation (DI) among patients with opiate use disorders who also carried a diagnosis of cellulitis.

Methods: A retrospective cross-sectional study was performed in one health system to identify patients with opiate use disorder and cellulitis over the past three years who also described symptoms of DI. We collected demographics, comorbid substance use disorder, and psychiatric diseases and calculated the likelihood of association with drug use by applying the Naranjo Adverse Drug Reaction scale.

Results: Fifteen patients (3.2% of all opiate-dependent individuals) were identified as also having DI. All patients had recurrent symptoms of DI, persisting over 18.3 ± 16 months with 4.6 ± 2.3 different health system contacts. The mean age was 44.4 years (range: 25 to 63 years). Twelve were female (80%, chi-square 5.4, p = 0.02). All had open wounds. Psychiatric comorbidities were common, but only two were schizophrenic, and none were actively psychotic during contact with the health system. Symptoms were either definitely (n = 1) or probably (n = 14) associated with drug exposure, according to the Naranjo Adverse Drug Reaction scale. Thirteen (87%) were also abusing other stimulants. All workups were negative for parasites and eosinophilia.

Conclusions: The prevalence of DI in patients with opiate use disorder was identified as significantly higher than reported in other populations. The role of DI in the formation of skin wounds deserves further study.

## Introduction

Opiate use disorder has evolved and accelerated in the United States over the past two decades, now affecting more than 2.1 million people with an estimated economic burden of $500 billion per year, including healthcare costs, lost productivity, and criminal justice expenses [1]. Since the mid to late 2010s, the illicit opiate market has transitioned from heroin to shorter-acting fentanyl [2]. Xylazine, an animal tranquilizer not approved for human use, had first been identified as an adulterant to fentanyl to improve euphoria and prolong the duration of injections as early as 2006 [2,3]. Fentanyl adulterated with xylazine (or FAAX) is now identified in more than 90% of illicit drug samples in Philadelphia in 2021 [2]. In addition to its acute effects, xylazine has been associated with severe necrotic skin ulcerations of unclear pathophysiology [4]. Over the last three years, our Street Medicine team, a homeless interventional outreach program, has observed an increase in open skin wounds along with symptoms consistent with delusional infestation (DI) in this cohort of patients. DI is an uncommon psychiatric disorder occurring in 27.3 per 100,000 person-years [5]. Despite the absence of evidence, patients with DI are convinced that they are infested with living or inanimate organisms, often accompanied by skin sensations [6]. DI is categorized into primary, where no external cause is identified, and secondary, which is associated with Parkinsonian drugs, antidepressants, and stimulant use, including methamphetamine and cocaine [6]. A case of DI secondary to heroin withdrawal has been reported in a single case report, but there are no published reports of DI with active opiate use [7]. Despite evidence linking xylazine adulteration to necrotic skin wounds, its potential role in DI among active opiate users remains unexplored, representing a critical knowledge gap with significant public health implications [4,5]. The aim of this study is to better understand the prevalence and characteristics of DI among patients with opiate use disorder who also carried a diagnosis of cellulitis.

## Materials and methods

Study design and setting

This was a single-center retrospective study at Tower Health, a six-hospital health system in the vicinity of Philadelphia, Pennsylvania. The study was approved by Tower Health’s Institutional Review Board (Protocol 2024_013_RH) in March 2024.

Selection of participants

A cohort of patients was identified in Epic, our electronic health record (EHR), to identify patients with SNOMED codes 160670007 for “substance abuse” and 80380006 for “cellulitis” from January 2021 to January 2024 using the EPIC Slicer-Dicer search function (Epic Systems, Verona, WI; Version: November 2023). Those subjects were then selected as adults (>18 years old) with positive urine drug screens for opiates, including fentanyl, methadone, and buprenorphine. A conservative approach was taken by excluding patients with a single episode of DI. Such cases were identified through a review of EHRs where DI symptoms were documented in only one health system encounter, based on provider notes and patient-reported symptoms. This method ensured a focus on persistent and clinically significant presentations, allowing for a more accurate estimation of the prevalence of persistent DI.

Data selection

The selected patients’ charts in the EHR were then searched by text word for the clinical features of DI, searching charts for keywords “bugs,” “worms,” “crawling,” and “infestation.” Each note in the EHR mentioning the clinical features was reviewed for significant DI symptoms. The duration and frequency of health system contacts were standardized by reviewing EHRs for encounter dates and visit documentation. Each contact was confirmed by the presence of provider notes or patient-reported symptoms of DI within the record, ensuring consistency and accuracy in data collection.

Analysis

Data analysis included two independent reviewers who determined the presence or absence of DI using the case definition of Freudenmann and Lepping (2009), who defined DI as a pervasive thought that the person is infested with either a pathogen-like insect or an inanimate object despite there being no medical or physical evidence to support their belief [8]. We selected cases in which patients demonstrated persistent (defined as more than one contact with the health system) delusions of an infestation without objective evidence of parasites. Patients with opiate use but no chart evidence of DI were counted in the denominator to determine the prevalence of DI in this population. Two reviewers independently screened the positive cases, and a third reviewer resolved disparities. The reviewers consisted of nurse practitioners and physicians with expertise in internal medicine, infectious disease, or both, ensuring a thorough and reliable evaluation of DI symptoms. Cohen’s kappa was calculated for agreement. Data extracted from charts included demographic information, number of contacts with complaints of DI, duration of contact, clinical features of the illness, including descriptions from patients, frequency of patients presenting with evidence of purported parasites (“baggie sign”), comorbid psychiatric diseases, comorbid drug use (defined as any drug use noted by the patient or in drug screens), open wounds (defined here as open wounds with exposed underlying tissue, either by report or by included image), comorbid alcohol and tobacco use, investigations for parasites including eosinophilia, and data to complete the Naranjo Adverse Drug Reaction (ADR) Probability Scale (Table 1) [9], a scoring system designed to estimate the likelihood that a reaction is related to a pharmacologic agent. The "baggie sign" refers to patients bringing samples of skin debris, hair, or other materials in a bag, believing they contain the parasites responsible for their symptoms, which is a recognized diagnostic clue in DI [8]. Any “Don’t know” answers for the Naranjo scale were scored as zero to calculate the most conservative estimate of the score. Chi-square goodness-of-fit testing was used to compare dichotomous variables. All statistical analyses were performed using IBM SPSS Statistics version 28 (IBM Corp, Armonk, NY), ensuring accurate and reproducible results. Two reviewers extracted data independently, with disparities adjudicated by a third reviewer. To mitigate potential biases introduced by the retrospective design, a standardized case definition was applied, and SNOMED codes were used to systematically identify relevant cases. This approach ensured consistency in data identification, while independent data extraction by reviewers further enhanced reliability.

**Table 1:** Naranjo Adverse Drug Reaction Probability Scale Interpretation: >9 = Definite; 5-8 = Probable; 1-4 = Possible; 0 = Doubtful.

Question	Yes	No	Do Not Know	Score
1. Are there previous conclusive reports on this reaction?	+1	0	0	
2. Did the adverse event appear after the suspected drug was administered?	+2	-1	0	
3. Did the adverse event improve when the drug was discontinued, or a specific antagonist was administered?	+1	0	0	
4. Did the adverse event reappear when the drug was readministered?	+2	-1	0	
5. Are there alternative causes that could on their own have caused the reaction?	-1	+2	0	
6. Did the reaction reappear when a placebo was given?	-1	+1	0	
7. Was the drug detected in blood or other fluids in concentrations known to be toxic?	+1	0	0	
8. Was the reaction more severe when the dose was increased or less severe when the dose was decreased?	+1	0	0	
9. Did the patient have a similar reaction to the same or similar drugs in any previous exposure?	+1	0	0	
10. Was the adverse event confirmed by any objective evidence?	+1	0	0	

## Results

The search of our EMR yielded 1,050 unique patients with a diagnosis of cellulitis and substance abuse over the three years of our study. Of these, 466 had a positive screen for opiates. Fifteen of the 466 patients (3.2%) met the study’s inclusion criteria for DI. There were no disagreements among reviewers for case inclusion. The agreement between observers for the extracted data was strong, with a Cohen’s kappa of 0.92. Based on standard thresholds, a kappa value exceeding 0.81 signifies near-perfect agreement. This high level of concordance highlights the reliability and consistency of the data extraction and diagnostic process.

Table 2 presents the clinical and demographic characteristics of the study participants. The mean age at diagnosis was 44.4 years (range: 25 to 63 years). The majority were female (12/15, 80%; chi-square = 5.4, p = 0.02). Psychiatric comorbidities were common, with anxiety, bipolar disorder, and depression being the most frequently observed conditions. However, only two participants had schizophrenia, and none were actively psychotic at the time of their interactions with the health system.

**Table 2:** Clinical and Demographic Characteristics of Patients Presenting With Suspected Delusional Infestation Sex: M = Male; F = Female. Psychiatric Comorbidities: A = Anxiety; B = Bipolar; D = Depression; S = Schizophrenia; P = Post-Traumatic Stress Disorder; O = Obsessive-Compulsive Disorder. Substance Use: F = Fentanyl; C = Cocaine; M = Methamphetamine. Naranjo ADR Probability Scale: Probable = score 5-8 (likely association); Definite = score ≥9 (highly certain association).

Age (Y)/Sex	Patient Complaints	Number of Health System Contacts	Duration of Episodes (Months)	Comorbid Psychiatric Diseases	Comorbid Substance Use	ADR Probability Scale
52/M	Reports "bugs of all different colors" crawling on him; picks at his skin constantly.	10	40	A, D	F, M	8 (Probable)
32/F	Patient reports wounds on arms and states "worms" are in the wounds.	4	3	A, D, P	F, C	9 (Definite)
49/F	Patient reports scabs on her body and feels something crawling through her skin.	7	13	A, B, S	F, M, C	8 (Probable)
56/F	Feels things crawling all over her; sees bugs in wounds and scratches to remove them.	5	31	A, B, S	F, M, C	8 (Probable)
64/F	Reports worms coming out of ears, eyes, hands, and feet.	4	12	D	F, M, C	8 (Probable)
51/F	Reports worms from arms, legs, and feet; small red spots enlarge with scratching.	3	7	B	F, M	8 (Probable)
39/F	Reports "bugs crawling on her skin."	3	18	A, B, D	F, C	8 (Probable)
32/F	Reports "bugs on her skin in her ear."	7	60	D	F, M	7 (Probable)
44/F	Feels bugs crawling through her skin; shaved her head and scratches her skin with tools.	8	14	A, D	F, M	7 (Probable)
43/F	Reports "bugs crawling on her" and carries a baggie with "bugs."	3	3	A, D	F, M, C	8 (Probable)
46/F	Endorses suicidal ideation if "bugs" do not go away.	5	24	B, O	F, M, C	8 (Probable)
62/F	Believes chronic pruritus is due to bugs.	3	37	B	F, M	8 (Probable)
54/F	Thinks bugs are crawling under her skin; believes maggots are in vomit.	2	5	B	F, M, C	8 (Probable)
43/F	Scratches itchy areas; sees white bugs coming out and asks for tweezers.	4	4	None	F	7 (Probable)
41/M	Reports "bugs crawling out of me."	2	4	A, D	F	7 (Probable)

All patients exhibited recurrent symptoms of DI, persisting over an average of 18.3 ± 16 months, with 4.6 ± 2.3 different health system contacts. According to the Naranjo ADR scale, one patient was definitively associated with drug exposure, while 14 were probably associated. Co-administration of other stimulants was common (13/15, 87%). Marijuana use was uncommon (3/15, 20%), and no hallucinogens were detected on drug screens. Nine patients consumed alcohol.

Two patients presented with plastic bags containing “specimens” thought to be parasites, but these were later identified as skin elements. All subjects had open wounds documented in their medical records through photographs or provider descriptions. Laboratory workups revealed no evidence of parasitic infection, and none of the patients had eosinophilia.

## Discussion

In this retrospective review of opiate-dependent individuals with DI, patients were predominantly female (74%), with an average age of 44.4 years, as compared to a large series by Foster et al. in which 80% were female, and the average age was 57 years. Foster and colleagues identified similarly high levels of comorbid psychiatric diagnoses (74% vs. 93% in our cohort), but our population was much less likely to be married (7%) than the group identified by Foster (56%) [10]. Our cohort of substance-dependent individuals had a significantly higher prevalence of DI (3.2%, or 3200 per 100,000) than has been reported elsewhere. In comparison, a population-based study among the residents of Olmsted County, Minnesota, from 1976 to 2010 found an incidence of DI of 1.9 cases per 100,000 person-years and age- and sex-adjusted prevalence of 27.3 per 100,000 years [11]. Our study focused on a more recent three-year period to capture current trends, such as the growing prevalence of xylazine-adulterated fentanyl in the drug supply. While this approach ensures relevance to clinical challenges, it may limit the ability to observe long-term trends. Population-based surveys in Germany have determined the incidence to be between 23.7 and 170 per 100,000 person-years, with a prevalence of 55.8 to 830 per 100,000 [8]. Our cohort of substance-dependent individuals had a significantly higher prevalence of DI (3.2%, or 3200 per 100,000) than has been reported elsewhere. This elevated prevalence can be partly attributed to the inclusion of a substance-dependent population, as substance use, particularly the co-abuse of stimulants like methamphetamines and cocaine, is associated with a higher risk of psychiatric symptoms, including DI [12,13]. These findings underscore the need for targeted interventions in similar high-risk populations to address the unique challenges posed by substance use and associated psychiatric conditions. Tactile hallucinations have been associated with active cocaine abuse (referred to as “cocaine bugs”) in numerous previous studies, with rates as high as 21% [12]. Psychosis is quite common in patients abusing methamphetamines, with rates of auditory and tactile hallucinations during active use as high as 40% [13]. The risk of psychosis is higher when there is polydrug use, other psychiatric disorders, and a family history of psychiatric disorders [13]. Although our cohort of patients with opiate abuse and symptoms of DI very often co-abused stimulants (87% had either cocaine or methamphetamines in at least one drug screen), this was the first report we could find on persistent DI symptoms in patients abusing fentanyl. Given the high rate of xylazine presence in the fentanyl supply, we hypothesize that this may potentially be the reason for our high prevalence of DI. The association between xylazine and DI may be linked to its pharmacologic effects as an alpha-2 adrenergic agonist, potentially exacerbating striatal dopamine transporter dysfunction, which is implicated in delusional parasitosis [14]. The interplay between xylazine and other stimulants, such as cocaine and methamphetamines, may amplify these effects, increasing the risk of persistent delusional symptoms and warranting further investigation [12]. The mechanism for xylazine-induced skin lesions is still currently not elucidated [15]. Interestingly, most reports show lesions in areas easily accessible to scratching and picking (forearms, lower legs) and rarely inaccessible areas (such as the back) [4]. The role of self-induced skin damage related to self-trauma associated with DI requires additional study, and whether DI prevalence is unique to this community or is more widespread will take further investigation [4]. The "baggie sign," observed in two patients who presented with plastic bags containing perceived parasites, highlights a key behavioral characteristic of individuals with DI. This phenomenon underscores the strength of the patients' convictions about their condition and presents a diagnostic challenge, as healthcare providers must navigate these strong beliefs while ruling out organic causes [8]. Given the connection between DI and stimulant use, the best course of action in treating a DI secondary to drug use would be cessation of the offending drug. Even though there is no clear data supporting the effectiveness of antipsychotic treatment, risperidone and pimozide are effective in systematic reviews and guidelines for the management of primary DI [16-20].

Limitations

This study has several limitations. The retrospective design limited the scope of evidence, and our inclusion criteria may have missed cases of early xylazine exposure without visible wounds. The use of specific SNOMED codes and unvalidated keywords may have excluded relevant cases or led to misclassification. Additionally, scoring "Don't know" answers as zero on the Naranjo scale may have underestimated the association between drug exposure and DI symptoms. The conservative inclusion criteria, requiring more than one documented episode of DI, and the population's lack of stable medical care likely led to an underestimation of the true prevalence, particularly among transient cases and those lost to follow-up. The study did not account for potential confounders like socioeconomic status or homelessness, which may have influenced DI prevalence. While psychiatric comorbidities were documented, their role in predisposing individuals to DI or worsening symptoms was not analyzed in depth. Reliance on clinical observations without validated biomarkers or confirmatory tests for xylazine exposure limits attributing DI symptoms directly to xylazine-adulterated fentanyl. Limited documentation of xylazine in physician notes may reflect insufficient awareness of its role, affecting diagnostic accuracy. Alternative mechanisms, such as peripheral sensory neuropathies, and comparisons of DI prevalence in substance use disorder populations versus the general population were not explored, warranting further research.

## Conclusions

This study identified a higher prevalence of DI among individuals with opiate use disorder and cellulitis compared to other populations. DI symptoms were strongly associated with substance use, particularly polydrug abuse, and unrelated to parasitic infections. The predominance of female patients and the chronicity of symptoms highlight the need for targeted interventions. The potential link between DI and necrotic skin wounds in the context of xylazine-adulterated opiates requires further investigation to improve management and outcomes.
